# Duplication of the Vas Deferens: A Rare Anomaly

**DOI:** 10.31486/toj.19.0114

**Published:** 2021

**Authors:** Muhammad Osama, Anosh Aslam Khan, Osama Mohiuddin, Choudhry Muhammad Saad, Shafaq Naseer, Farhan Zaheer

**Affiliations:** ^1^ Department of General Surgery, Dow University of Health Sciences, Karachi, Pakistan; ^2^ Department of Internal Medicine, Dow University of Health Sciences, Karachi, Pakistan

**Keywords:** *Herniorrhaphy*, *spermatic cord*, *vas deferens*

## Abstract

**Background:** Duplication of the vas deferens, a rare congenital anomaly of the pelvic anatomy, is often an incidental finding during surgeries involving the spermatic cord, such as inguinal hernia repair, varicocelectomy, orchidopexy, and vasectomy.

**Case Report:** A 25-year-old male presented to our surgical outpatient clinic with bilateral swelling in the inguinal region. A diagnosis of bilateral inguinal hernia was established. While performing spermatic cord dissection during hernioplasty, a duplicated vas deferens was revealed within the left spermatic cord. Doppler ultrasonography confirmed the absence of waveforms in both vasa deferentia, differentiating them from adjacent vessels. The hernia repair was performed without complications.

**Conclusion:** Our case highlights the importance of radiologists’ and surgeons’ ability to recognize a duplicated vas deferens to avoid possible iatrogenic injury.

## INTRODUCTION

Duplication of the vas deferens is a rare congenital anomaly of the male reproductive system, with an estimated incidence of approximately 0.05%.^1^ Vas deferens duplication is often an incidental finding during surgeries involving the spermatic cord, such as inguinal hernia repair, varicocelectomy, orchidopexy, and vasectomy.^2^

Embryologically, the proximal vas precursor is a segment along the mesonephric duct, located at an intermediate position between the upper and common mesonephric ducts. The proximal vas precursor differentiates into the vas deferens and seminal vesicles, while the common mesonephric duct interacts with the metanephric blastema and develops into the kidney and its collecting system. The duplication of the proximal vas precursor leads to duplication of the vas deferens at the level of the inguinal canal. A double vas deferens, an ectopic ureter draining into the ejaculatory system, is sometimes mistakenly called a duplicated vas deferens.^1-3^

Recognition of a duplicated vas deferens is vital to prevent trauma and iatrogenic injury during surgical procedures. We present the case of a young male diagnosed with bilateral inguinal hernia who was found to have a duplicated vas deferens.

## CASE REPORT

A 25-year-old male with no known comorbidities presented to our surgical outpatient clinic with painless bilateral swelling in the inguinal region for 2 years. The swelling became prominent with walking and disappeared when the patient was lying down. He denied any urinary or abdominal symptoms. Medical, surgical, and family histories were not significant.

On examination, no swelling was apparent in the inguinoscrotal region bilaterally with the patient in either standing or supine position. Reexamination after the patient had walked for 10 minutes revealed bilateral swelling in the inguinoscrotal region that was reducible, nonadherent, and approximately 2 × 3 cm in size. The swelling did not extend to the scrotal base; it was located above and medial to the pubic tubercle. Cough impulse was positive bilaterally, while the ring occlusion test was only positive on the left side. The transillumination test was unremarkable on either side. The patient had no evidence of any other swelling or vascular engorgement. Both of the spermatic cords and testicles were normal upon examination.

Basic presurgical workup—including baseline investigation and cardiac and anesthesia review—was normal. Bilateral inguinoscrotal ultrasound findings suggested bilateral indirect inguinal hernias. Ultrasound of the whole abdomen did not show any renal abnormalities.

The patient underwent open bilateral inguinal hernioplasty via the Lichtenstein technique under spinal anesthesia. During dissection of the right groin, neither a direct nor an indirect hernia sac was identified; no evidence of hernia was found in the right groin region. Instead, a small-sized cord lipoma was found and carefully excised. During exploration of the left inguinal region, an indirect, thin-walled hernia sac was noted. Dissection of the hernia sac from the adjacent cord structure revealed 2 separate vasa deferentia that were positioned anteromedially to the hernia sac (Figure). Both vasa deferentia were observed to be draining into the right testicle, an observation confirmed on palpation. Drainage of each vas deferens into the testicle was not assessed visually as the testis was in the scrotal sac and was not retracted from the scrotum unnecessarily. Perioperative examination of the left scrotum showed a single, normally located testicle and epididymis. Caudally, both vasa deferentia were communicating separately with the tail of the epididymis. Intraoperative Doppler confirmed this anomaly. Neither of the structures exhibited any waveform signal. However, a prominent waveform signal was observed in the artery adjacent to the vasa deferentia, confirming the viability of both vasa deferentia. Caution was taken to keep both vasa deferentia intact. When the hernia sac was opened, it was found to be empty and was closed using purse-string sutures. Prolene mesh measuring 6 × 11 cm was sutured in place. The patient tolerated the procedure well.

**Figure.**
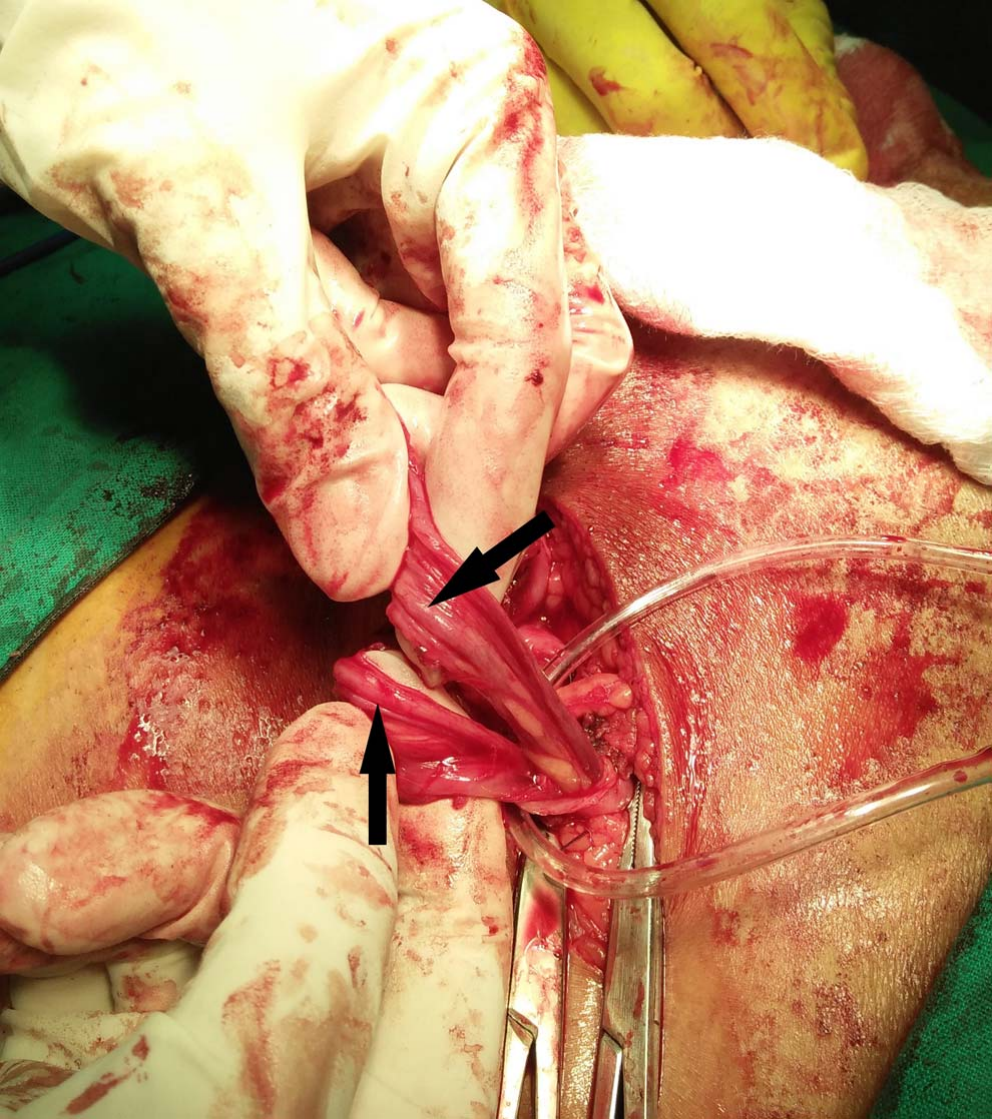
**Duplicated vas deferens emerging from a single testicle during spermatic cord dissection.**

Postoperative ultrasound of the whole abdomen and inguinoscrotal region was normal, the same as the preoperative ultrasound, and did not reveal any postoperative complications such as recurrence of hernia or hematoma. The patient had no scrotal edema or urinary retention.

The patient was discharged the day after surgery with no postoperative complications. At 3-month follow-up, he did not report any fertility issues or masses in the region of the surgery. His semen analysis was within normal limits.

## DISCUSSION

The vas deferens, also known as the ductus deferens, is the part of the male reproductive system that transports sperm from the epididymis to the ejaculatory duct. Anatomic variations of the vas deferens include absence, duplication, ectopia, hypoplasia, and diverticulum, with true duplication the rarest congenital anomaly. Although vas deferens duplication is generally an isolated occurrence, it can also be present with other congenital abnormalities such as ipsilateral renal agenesis or cystic fibrosis, but no cases of associated genitourinary system abnormalities have been reported.^4^

Liang et al introduced a classification system for vas deferens anomalies^2^:

- Type I: Classic duplicated vas deferens (complete or partial) in a spermatic cord with no polyorchidism
- Type II: Multiple vasa deferentia associated with polyorchidism
- Type III: Double vas deferens composed of an ectopic ureter ending in the ejaculatory system

Duplication of the vas deferens is difficult to diagnose on physical examination because of its infrequent occurrence. Pathologic evaluation, imaging, and histologic examination can enhance clinical acumen. Cases of duplicated vas deferens often entail urology consultations for confirmation. Intraoperative Doppler helps to differentiate the vas deferens from surrounding vasculature which can prevent iatrogenic injury.^5^

Considering the high prevalence of surgeries involving the spermatic cord and neighboring structures, duplication of the vas deferens may be underdocumented and underrecognized. Associated iatrogenic injuries include scarring of the vas deferens, formation of sperm granuloma, and chronic pain.^6^ However, the most serious medicolegal complication is infertility, mandating prompt reexploration in suspected cases.^7^ Spermatic granuloma can develop after extravasation of sperm from an injured vas deferens. Spermatozoa are highly antigenic and trigger an inflammatory reaction that eventually forms a nodule surrounding the defect, resulting in postoperative groin pain and requiring anastomosis of the severed vas deferens.^8^ Duplication of the vas deferens is also associated with unsuccessful vasectomies, prompting readmission and resterilization to achieve the desired outcome.^9^

Ultrasound of the whole abdomen and genitourinary tract should be performed to eliminate the possibility of genitourinary tract anomalies such testicular ectopia and renal agenesis.^2^ Ultrasonography has more than 95% sensitivity in detecting renal anomalies^10^ and can also be used to rule out postoperative complications of laparoscopic hernia repair such as recurrence of hernia, hematoma, and abscess.^11^

Although our patient did not experience any surgical trauma or postsurgical complications, other studies and case reports have indicated otherwise. In a study by Sheynkin et al, 7.2% of 472 patients had iatrogenic injury to the vas deferens.^6^ Silich and McSherry described the gradual development of spermatic granuloma because of erosion of the vas deferens wall by the cut margin of mesh placed during hernia repair.^8^ In a case reported by Carr, postsurgical semen analysis after vasectomy detected spermatozoa for several months.^12^ Surgeons must be aware of this anatomic variant to reduce the risk of injury and postsurgical adverse results, as the rate of hernia repair surgeries annually is high worldwide.^13^

## CONCLUSION

Because duplicated vas deferens is an asymptomatic anatomic variant, radiologists, surgeons, and urologists must recognize and appreciate this rare anomaly. Preoperative radiographic imaging can provide guidance for surgeons for careful preservation of the structure. Routine identification and reporting of duplication of vas deferens can help to spread awareness of the condition among surgeons and avoid postoperative complications in patients.

## Notes

This article meets the Accreditation Council for Graduate Medical Education and the American Board of Medical Specialties Maintenance of Certification competencies for Patient Care and Medical Knowledge.
